# Reference Intervals of Thyroid Hormones and Correlation of BMI with Thyroid Function in Healthy Zhuang Ethnic Pregnant Women

**DOI:** 10.1155/2018/2032413

**Published:** 2018-11-14

**Authors:** Yonghong Sheng, Dongping Huang, Shun Liu, Xuefeng Guo, Jiehua Chen, Yantao Shao, Guoqiang Zhang, Liangjia Wei, Xiaoyun Zeng, Xiaoqiang Qiu

**Affiliations:** ^1^Department of Epidemiology and Health Statistics, School of Public Health, Guangxi Medical University, Nanning 530021, Guangxi, China; ^2^Department of Sanitary Chemistry, School of Public Health, Guangxi Medical University, Nanning 530021, Guangxi, China

## Abstract

Ethnic differences in the level of thyroid hormones exist among individuals. The American Thyroid Association (ATA) recommends that an institution or region should establish a specific thyroid hormone reference value for each stage of pregnancy. To date, a limited number of studies have reported the level of thyroid hormones in Chinese minorities, and the exact relationship between BMI and thyroid function in pregnant women is ill. This study was performed to establish trimester-specific reference ranges of thyroid hormones in Zhuang ethnic pregnant women and explore the role of body mass index (BMI) on thyroid function. A total of 3324 Zhuang ethnic health pregnant women were recruited in this Zhuang population-based retrospective cross-sectional study. The values of thyroid stimulating hormone (TSH), free thyroxine (FT4), and free triiodothyronine (FT3) were determined by automatic chemiluminescence immunoassay analyzer. Multivariate linear regression and binary logistic regression were constructed to evaluate the influence of BMI on the thyroid function. The established reference intervals for the serum thyroid hormones in three trimesters were as follows: TSH, 0.02–3.28, 0.03–3.22, and 0.08-3.71 mIU/L; FT4, 10.57–19.76, 10.05–19.23, and 8.96–17.75 pmol/L; FT3, 3.51–5.64, 3.42–5.42, and 2.93–5.03 pmol/L. These values were markedly lower than those provided by the manufacturers for nonpregnant adults which can potentially result in 6.10% to 19.73% misclassification in Zhuang pregnant women. Moreover, BMI was positively correlated with isolated hypothyroxinemia (OR=1.081, 95% CI=1.007–1.161), while the correlation between the BMI and subclinical hypothyroidism was not statistically significant (OR=0.991, 95% CI=0.917–1.072). This is the first study focusing on the reference ranges of thyroid hormones in Guangxi Zhuang ethnic pregnant women, which will improve the care of them in the diagnosis and treatment. We also found that high BMI was positively associated with the risk of isolated hypothyroxinemia.

## 1. Introduction

A number of adverse outcomes of thyroid dysfunction on pregnant women and offspring have long been established. For example, elevated maternal thyroid stimulating hormone (TSH) has been associated with an increased risk of preterm delivery, miscarriage, and fetal demise [1, 2]. Overt and subclinical hypothyroidism and overt hyperthyroidism have been associated with a risk of intrauterine growth restriction [3, 4]. The incidence of gestational diabetes mellitus and preeclampsia were negatively correlated with maternal free thyroxine (FT4) levels [5]. Also confusing is the level of thyroid hormones during pregnancy can change markedly and show a significant difference compared with those of nonpregnant people [6, 7]. The 2017 Guidelines of the American Thyroid Association (ATA) for the Diagnosis and Management of Thyroid Disease during Pregnancy and the Postpartum have detailed the influence of increase in renal iodine excretion, thyroxine binding proteins, thyroid hormone production, and human chorionic gonadotropin (hCG) on thyroid function in the pregnant women [8]. With a similar structure and activity to TSH, hCG can directly stimulate the secretion of FT4 and FT3 and inhibit the production of TSH through a feedback pathway [9]. The demand for iodine in fetus and the urine iodine elimination of pregnant women increase in later trimesters. This condition would lead to a relative lack of iodine in pregnant women and cause thyroid dysfunction [10]. If thyroid hormone reference values for nonpregnant women were applied to pregnant women, it would lead to 5.6% to 18.3% misdiagnosis or missed diagnosis of thyroid disease [11]. Moreover, variations in the thyroid hormones were observed in different ethnicity or regions probably because of the differences in genetic susceptibility caused by polymorphisms or differences in environmental exposures [12, 13]. A significant decrease in the median TSH levels was found in African-Americans compared with Caucasians in the National Health and Nutrition Examination Survey III report [14]. Ho* et al*. demonstrated that the upper reference limit of the total T3 was higher in Malays than in Chinese in a multiethnic population [15]. The decline of TSH level happened in essentially almost of pregnant women. In the 2017 Guidelines, the ATA pointed out that the extent of TSH reduction varies significantly between different racial and ethnic groups and recommended that each institution or region should establish their own population-based and trimester-specific reference ranges [8]. However, in China, most of the reference values of thyroid hormones currently used for pregnant women are provided by foreign assay manufacturer for normal adults [7]. And there is no specialized reference data applied in Zhuang people. For these reasons, it is needed to establish a specialized reference data for Zhuang pregnant women.

Several studies have demonstrated that the pregnant women's BMI may have an impact on the thyroid function. TSH levels increase with BMI, but FT4 levels decrease with increasing BMI [16]. Han* et al*. reported that high BMI was an indicator of hypothyroidism, hypothyroxinemia, and thyroid-peroxidase antibody positivity in Chinese pregnant women [17, 18]. In 2011, the ATA proposed that the level of TSH should be specifically detected in pregnant women with abnormal obesity [19]. By contrast, Haddow and Pop found no relation between the BMI and serum TSH levels in any trimesters in healthy pregnant women [20, 21]. Hence, the relationship between the BMI and thyroid function in pregnant women remains controversial.

The Zhuang ethnic minority is China's largest minority group with more than 16 million population, and most of the Zhuangs live in the remote mountainous regions in Guangxi. Jingxi City and Longan County were selected, because they were typical gathering areas of the Zhuang population. This study was performed to calculate the gestational age-specific reference intervals of thyroid hormones and explore the effect of BMI on the thyroid function in Zhuang ethnic pregnant women.

## 2. Materials and Methods

### 2.1. Subjects

**Figure 1:**
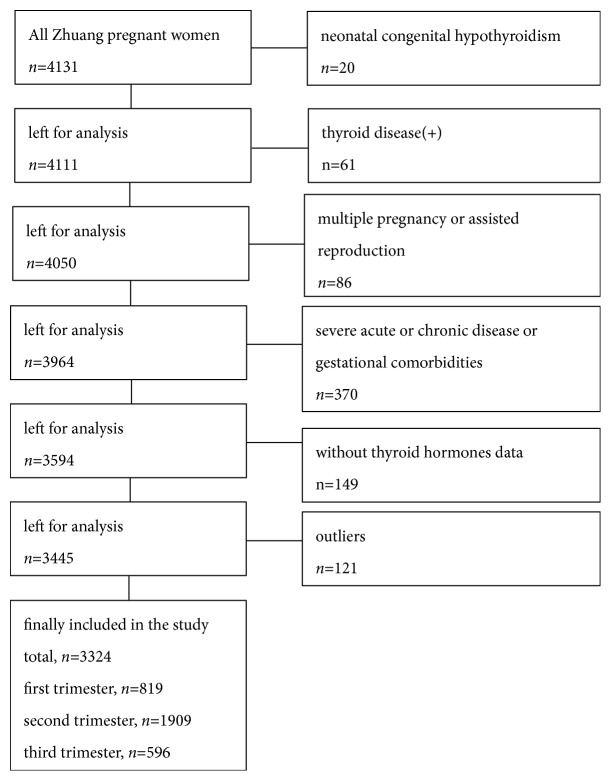
Flowchart of included/excluded Zhuang ethnic pregnant women.

A total of 4131 Zhuang ethnic pregnant women aged 18–45 years were recruited in the People's Hospital of Jingxi and Longan from November 2016 to May 2018. All pregnant women had regular checkups at the hospital. Based on the guidelines issued by the American Academy of Clinical Biochemistry [22], the exclusion criteria were as follows: had newborns who suffered from neonatal congenital hypothyroidism; personal history or family history of thyroid disease, goiter, and autoimmune diseases; administered drugs that affect thyroid function (amiodarone, estrogen, glucocorticoids, and antiepileptic drugs); with serious acute/chronic diseases or pregnancy complications (hypertension, diabetes, liver disease, renal disease and mental disorder); multiple pregnancy; assisted reproduction; without thyroid hormone data; and outliers. The outliers were detected by the horn algorithm (with a ratio of 2.2) [23]. Eventually, 807 were excluded, resulting in a final sample of 3324 healthy pregnant women analyzed according to the following trimesters: first, 819 cases; second, 1909 cases; and third, 596 cases. The main flowchart of included/excluded pregnant women is shown in Figure 1.

### 2.2. Data Collection

The face-to-face questionnaire conducted by obstetricians covered maternal general demography, pregnancy history, mother/family medical history, and health behavior during pregnancy and perinatal period. Blood samples were collected in the morning after fasting or a light meal and centrifuged after complete clotting. All serum samples were detected by a laboratory physician within 3 h by automatic chemiluminescence immunoassay analyzer (ADVIA Cencaur XP, SIEMENS, USA) using reagent kits from Siemens. The limits of quantification were 0.01–100 mIU/L, 0–100 pmol/L, and 0–30 pmol/L for TSH, FT4, and FT3, respectively. The reference ranges for adults provided by the manufacturers were 0.55–4.78 mIU/L, 11.5–22.7 pmol/L, and 3.5–6.5 pmol/L for TSH, FT4, and FT3, respectively.

### 2.3. Study Variables

The gestational age was calculated based on the women's certain last menstrual period (LMP). Sonography was performed if discrepancies existed between the fundal height and LMP or if the LMP was uncertain. The gestational age was classified into the following trimesters: first trimester, gestational age <13 weeks; second trimester, gestational age of 13–28 weeks; and third trimester, gestational age ≥ 28 weeks. The BMI was assessed with TSH, FT4, and FT3 at the same trimester. This index was calculated and expressed as weight (kg)/height (m^2^). According to the Chinese body mass characteristics, the BMI was divided into underweight (BMI<18.5 kg/m^2^), normal weight (BMI=18.5–24 kg/m^2^), overweight (BMI=24–28 kg/m^2^), and obese (BMI≥28 kg/m^2^). The Endocrine Society and ATA guidelines recommended the usage of population-based trimester-specific reference ranges as the diagnostic standard of thyroid function [19]. The thyroid function was classified into euthyroid, subclinical hypothyroidism, and isolated hypothyroxinemia. Women with serum TSH and FT4 within the trimester-specific reference ranges were considered to be euthyroid. TSH values above the 97.5th percentile and with a normal FT4 concentration were identified with subclinical hypothyroidism. Isolated hypothyroxinemia was defined as a normal maternal TSH concentration in conjunction with FT4 concentrations in the lower 2.5th percentile of the trimester-specific reference range. We did not analyze the overt types because of the small sample size within the group.

### 2.4. Statistical Analysis

Data were stored in a Microsoft Excel database and statistically analyzed using the SPSS version 23.0 software (SPSS Inc., Chicago, IL, USA). Continuous data were expressed as mean±SD for normally distributed data or median (IQR) when not normally distributed. The reference intervals were presented as medians (50th percentile) and the range of reference (95% range between the 2.5th and the 97.5th percentiles) in each trimester, and BMI, TSH, FT4, and FT3 were the nonnormal distribution (*p*<0.05 in the Kolmogorov-Smirnov test). The Kruskal-Wallis test was used for the comparison of median serum hormone levels among groups. The Mann–Whitney U test was used for pairwise comparisons. Multiple linear regression and binary logistic regression models were used to analyze the effect of BMI on the thyroid function. Two-sided* P* value of <0.05 was considered statistically significant.

### 2.5. Ethics Statement

This study was approved by the Ethics Committee of Guangxi Medical University and performed in accordance with the approved guidelines. Before participating, all participants signed an informed consent form.

## 3. Results

### 3.1. Characteristics of the Study Population

The study population consisted of 3324 pregnant women. Approximately 90% of these women lived in rural areas (*n*=2992). The mean age of the participants was 28.59±5.78 years. No significant differences were found in the ages and residences (rural, urban) among the different trimesters. The mean gestational periods for the first, second, and third trimesters were 10.99, 17.64, and 35.11 weeks, respectively, and the corresponding mean BMIs were 20.70, 22.48, and 25.51 kg/m^2^. Approximately one-third of these pregnant women were primipara (*n*=1073), while 67.72% were multipara (*n*=2251).

### 3.2. Reference Ranges for TSH, FT4, and FT3 according to Trimesters

**Figure 2:**
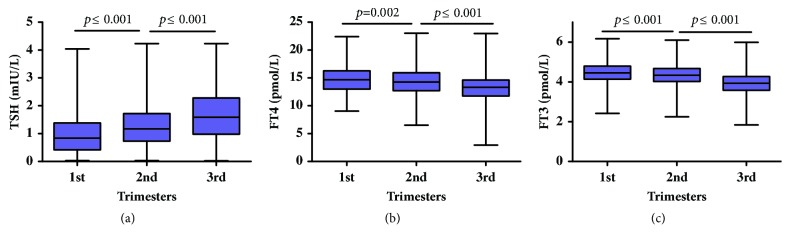
Box plot of TSH (a), FT4 (b), and FT3 (c) concentrations in the different trimesters.

The level of TSH was lowest in the first trimester and highest in the third trimester (medians for the first, second, and third trimesters: 0.84, 1.17, and 1.59 mIU/L, respectively;* p* ≤0.001 Kruskal-Wallis test). The differences in the TSH concentrations in each trimester were statistically significant (*p*≤0.001 Mann–Whitney U test) and ranged from 0.02 mIU/L to 3.71 mIU/L in all trimesters. Contrary to TSH, the highest and lowest levels of FT4 and FT3 were found in the first and third trimesters, respectively (FT4 medians for the first, second, and third trimesters: 14.67, 14.29, and 13.31 pmol/L;* p*≤0.001 Kruskal-Wallis test; FT3 medians for the first, second, and third trimesters: 4.45, 4.33, and 3.93 pmol/L, respectively;* p*≤0.001 Kruskal-Wallis test). The concentrations of FT4 and FT3 in each trimester were also significantly different (*p*<0.05 Mann–Whitney U test) (Table 1 and Figure 2).

### 3.3. Potential for Misclassification of Thyroid Function in Zhuang Pregnant Women if Nonpregnant Reference Intervals Are Used

For TSH, a total of 642(19.73%) Zhuang ethnic pregnant women would be potentially misclassified if using the nonpregnant reference intervals provided by the assay manufacturer. Of which, 562 (17.27%) women would have been incorrectly classified as having a low TSH, and 80 (2.46%) women with elevated TSH would not have been identified. Potential for misclassification of TSH results was greatest in the first trimester (32.38%). For FT4, 269 (8.25%) women would have been incorrectly classified as having a low FT4, and 78(2.39%) women with elevated FT4 would not have been identified. Potential for misclassification of FT4 results was greatest in the first trimester (19.97%). For FT3, 120 (3.68%) women would have been incorrectly identified as low, and 79 (2.42%) women would not have been identified as high. Potential for misclassification was greatest in the third trimester (20.28%) (Table 2).

### 3.4. Reference Ranges for TSH, FT4, and FT3 according to the BMI Categories in the Different Trimesters

The median concentrations of TSH significantly increased with BMI in the first trimester (*p*<0.05 Kruskal-Wallis test) but did not differ significantly in the second and third trimesters (*p*>0.05). The median concentrations of FT4 significantly decreased with increasing BMI in the second and third trimesters (*p*<0.05) but did not significantly differ in the first trimester (*p*>0.05). The level of FT3 increased with BMI in the first and second trimesters (*p*<0.05) but did not significantly differ in the third trimester (*p*>0.05) (Table 3).

### 3.5. Linear Trend Analysis between BMI and TSH, FT4, and FT3

In the multivariate linear model, TSH and BMI showed a significant linear trend in the first trimester (*p* for trend <0.05). The level of TSH was higher in overweight or obese pregnant women than in normal weight women (*β*=0.185, 95% CI: 0.026–0.345). However, no linear trend was found in the second and third trimesters (*p* for trend >0.05). FT4 and BMI showed a negative linear trend in the second and third trimesters (*p* for trend <0.05). FT4 concentration was significantly lower in overweight or obese pregnant women than in normal weight women [second trimester of overweight or obese *β*= −0.341, 95% CI: −0.578–0.104; third trimester of overweight *β*= −0.419, 95%CI: −0.829–(−)0.008; third trimester of obese *β*= −1.040, 95%CI: −1.558–(−)0.522]. FT3 and BMI showed a significantly positive linear trend in the first and second trimesters (*p* for trend <0.05). The concentration of FT3 was higher in overweight or obese pregnant women than in normal weight women (first trimester *β*=0.145, 95% CI: 0.040–0.251; second trimester *β*=0.132, 95% CI: 0.081–0.182) (Table 4).

### 3.6. BMI and Risk of Thyroid Dysfunction in Logistic Regression Analysis

After adjusting the residence type, age, gestational age, and parity, BMI was positively associated with isolated hypothyroxinemia (OR=1.081, 95% CI=1.007−1.161). No statistical difference was found between BMI and subclinical hypothyroidism (OR=0.991, 95%CI=0.917–1.072) (Table 5).

## 4. Discussion

We first recruited a large study of Zhuang ethnic pregnant women to investigate the gestational age-special reference intervals for thyroid hormones and explored the effect of BMI on thyroid function.

In our study, the 95% reference ranges of TSH, FT4, and FT3 in Zhuang pregnant women were markedly lower than the reference intervals for nonpregnant adults provided by the assay manufacturer. Interpretation of TSH, FT4 and FT3 in Zhuang pregnant women using nonpregnant reference intervals could potentially result in 6.10% to 19.73% misclassification. Our data demonstrated that the application of nonpregnant reference intervals in Zhuang pregnant women was particularly problematic, and supported the need for gestational age-specific reference intervals for Zhuang pregnant women. This finding is consistent with the recommendations from the ATA and the European Thyroid Association (ETA); that is, reference intervals specific for trimesters should be established and applied [19, 24]. Similar results were obtained in other studies [7, 19, 24]. The distinct thyroid hormone levels between pregnant women and nonpregnant people are mainly related to the rapid increase in the level of hCG, thyroid-binding globulin, estrogens, plasma volume, and relative iodine deficiency during pregnancy [8, 25].

If the reference intervals are not available from the local laboratory, in the 2011 guidelines, ATA has recommended the reference ranges for TSH of 0.1–2.5, 0.2–3.0, and 0.3–3.5 mIU/L for the first, second, and third trimesters, respectively [19]. In the present study, the lower limits of TSH were markedly lower, but the upper limits of TSH were slightly higher than recommended by the ATA guidelines in the three trimesters of pregnancy. Compared with a Korean study using the same TSH assay, the results showed close lower reference limits for TSH (0.01, 0.01, and 0.15 mIU/L in the first, second, and third trimesters, respectively), but the upper reference limits were lower [26]. At the same time, compared with the West Chinese pregnant women, the lower reference limits for TSH in the three trimesters were markedly lower than their reference limits (0.05, 0.61, and 0.65 mIU/L, respectively) [27]. The different results could be caused by the characteristics of the study population, methods of reference interval estimation, ethnic, sample size, and iodine status.

The current study showed that the highest FT4 and FT3 levels were observed in the first trimester and decreased in subsequent trimesters. These results are in line with the observations in other studies [28]. Our reference intervals for FT4 in the three trimesters were close to the reference ranges for Polish women (11.99–21.89, 10.46–16.67, and 8.96–17.23 pmol/L, respectively) [29] but wider than the reference intervals in the pregnant women of West China (12.29–18.92, 10.97–15.49, and 9.49–16.25 pmol/L, respectively) [27]. Our reference intervals for FT3 were similar to those of Kosteckamatyja, who used the same assay. Kosteckamatyja* et al*. determined that the trimester-specific reference intervals for FT3 of Polish pregnant women were in the ranges of 3.6–6.5, 3.3–5.5, and 3.1–5.4 pmol/L in the first, second, and third trimesters, respectively [29]. The differences in the reference intervals were probably due to diversity in several factors, such as measurement techniques, ethnic, sample size, selection criteria, sample processing, population-specific characteristics, and variation in iodine intake.

A positive linear trend was found between BMI and TSH level in the first trimester, but no relationships were found in the second and third trimesters. No correlation was found between BMI and subclinical hypothyroidism in the binary logistic regression models. The positive correlation between BMI and TSH level in the early trimester is consistent with the studies by Mannisto* et al*. and Bestwick* et al*. [22, 30]. Similarly, Han* et al*. found that the TSH level was significantly higher in overweight women than in normal weight women during early pregnancy, and no relationship was found between BMI and subclinical hypothyroidism [17]. Contrary to our study, Pop* et al*. determined no relationship between BMI and TSH level during the first trimester [20]. Haddow* et al*. also did not find any association between BMI and TSH in 9351 pregnant women from 11 to 20 gestational weeks [21]. The exact relationship between BMI and TSH remains ambiguous in pregnant women. Several mechanisms have been suggested. Leptin, produced by adipose tissue, has been shown to directly affect the thyroid axis at the hypothalamic and pituitary central through the stimulation of thyrotropin-releasing hormone [31]. Tim's study proposed that hCG and BMI have separate roles in this events [32]. And the relationship between BMI and TSH can be interfered by hCG, because hCG plays a feedback inhibition on TSH during the whole duration of pregnancy [33].

Another interesting result was the significant and inverse correlation of BMI with FT4 levels in the second and third trimesters, and high BMI is a risk factor to isolated hypothyroxinemia. Three related studies showed similar results [20, 34, 35]. For example, Pop showed that, in healthy Dutch Caucasian pregnant women, high BMI was related to lower FT4 levels throughout the whole gestation. Haddow* et al*. and Ashoor* et al.* detected an inverse relationship btween BMI and FT4 in pregnant women. Moreover, in nonpregnant population, Shon* et al*. and Marwaha* et al*. also demonstrated that FT4 was negatively associated with BMI. Shon* et al*. observed a negative association between FT4 and BMI in euthyroid women (mean age of 46.2 years) [36]. Marwaha showed that FT4 levels decreased with increasing BMI in euthyroid boys and girls [37]. Fernanda* et al*. found lower maternal FT4 level in early pregnancy is associated with a higher maternal prepregnancy BMI, but no association with mid and late-pregnancy BMI [38]. This may be caused by difference in trimester category and the fact that FT4 levels of participants were measured only in early pregnancy. The inverse association between BMI and FT4 is well established. Biologically, the stimulatory effect of FT4 on the metabolic rate could explain this relationship.

Contrary to the correlations of FT4 levels, FT3 concentrations showed a tendency to be positively associated with BMI. Our finding is consistent with the result obtained by Mannistoet, who observed positive association between FT3 and BMI in 5072 pregnant women [22]. Moreover, positive association between FT3 and BMI has been observed in a more general population [37, 39]. Several possible mechanisms have been proposed to explain these findings. T3 plays an important role in increasing energy expenditure and thermogenesis in order to keep in parallel with increases in body weight [40]. Weight loss is associated with a decrease in FT3 serum levels [41]. FT3 may be an adaptive mechanism for the increase in central fat accumulation. Leptin also apparently affects the level of FT3 [42].

This study is the first to calculate the gestational age-specific reference intervals of thyroid hormones for Zhuang ethnic pregnant women of China. Moreover, fresh serum samples were used in this study, because fresh serum sampling is optimal in creating laboratory reference intervals. However, this study has several limitations. First, the thyroid function could not be measured consecutively in each individual, and interindividual variation could exist. Second, the cross-sectional study design did not permit the detection of causality. Furthermore, we did not have data on thyroid autoantibodies, although the outliers of thyroid hormones have been excluded. Thyroid autoantibodies inclusion may have been beneficial to confirm the effect of BMI on thyroid function.

## 5. Conclusions

In summary, our data shows that the application of nonpregnant reference intervals has the potential to result in 6.10% to 19.73% misclassification in Zhuang pregnant women. We established reference intervals for TSH, FT4, and FT3 in Zhuang ethnic pregnant women and the results will facilitate the thyroid dysfunction screening in Zhuang ethnic pregnancy women. Furthermore, this study provides new evidence that high BMI may be a risk factor for isolated hypothyroxinemia and increased FT3 but not a risk factor for subclinical hypothyroidism in pregnant women.

## Figures and Tables

**Table 1:** The reference ranges for TSH, FT4, and FT3 in three trimesters of Zhuang ethnic pregnant women.

Trimesters	*n*	TSH(mIU/L)	FT4(pmol/L)	FT3(pmol/L)
1st	819	0.84 (0.02-3.28)	14.67 (10.57-19.76)	4.45 (3.51-5.64)

2nd	1909	1.17 (0.03-3.22)*∗*	14.29 (10.05-19.23)*∗*	4.33 (3.42-5.42)*∗*

3rd	596	1.59 (0.08-3.71)*∗*^#^	13.31 (8.96-17.75)*∗*^#^	3.93 (2.93-5.03)*∗*^#^

Manufacturers		0.55-4.78	11.50-22.70	3.50-6.50

The TSH, FT4, and FT3 levels were expressed as median and reference interval (2.5 and 97.5 percentiles) since they were nonnormal distribution, Kolmogorov-Smirnov test.*∗*Compared with 1st trimester, *p *< 0.05; ^#^compared with 2nd trimester, *p *< 0.05, Mann–Whitney U test.

**Table 2:** The number and percentage of results potentially misclassified if nonpregnant reference intervals are used.

		1st trimester		2nd trimester		3rd trimester		
	*N*	Misdiagnosis	Missed	Misdiagnosis	Missed	Misdiagnosis	Missed	Overall
TSH	3254	241(30.01%)	19(2.37%)	271(14.50%)	47(2.51%)	47(8.06%)	14(2.41%)	642(19.73%)

FT4	3260	29(3.54%)	20(2.44%)	137(7.37%)	45(2.42%)	103(17.73%)	13(2.24%)	347(10.64%)

FT3	3261	2(0.24%)	20(2.45%)	14(0.75%)	45(2.42%)	104(17.87%)	14(2.41%)	199(6.10%)

N: the number of women with TSH, FT4, and FT3 levels within 95% confidence intervals or within the reference intervals provided by the assay manufacture. Misdiagnosis: the level of thyroid hormones above 2.5th confidence limit in the present study and below the lower reference interval provided by the assay manufacture; missed: the level of thyroid hormones above 97.5th confidence limit and below the higher reference interval provided by the assay manufacture or the level of thyroid hormones above the lower reference interval provided by the assay manufacture and below the 2.5th confidence limit.

**Table 3:** The reference ranges for TSH, FT4, and FT3 according to BMI categories.

Trimesters	BMI(kg/m^2^)	*n*	TSH(mIU/L)	FT4(pmol/L)	FT3(pmol/L)
1st	<18.5	188	0.81 (0.01-3.47)	15.03 (10.85-19.72)	4.43 (3.35-5.67)

	18.5-24	511	0.84 (0.02-3.27)	14.57 (10.61-19.77)	4.42 (3.54-5.66)

	≥24	120	1.00 (0.05-3.26)	14.11 (9.92-19.78)	4.63 (3.50-5.73)

	*p *value		0.021*∗*	0.085	0.004*∗*

2nd	<18.5	104	1.25 (0.02-3.42)	15.13 (11.58-19.23)	4.22 (3.00-5.62)

	18.5-24	1276	1.17 (0.03-3.24)	14.47 (10.16-19.26)	4.31 (3.41-5.37)

	≥24	529	1.15 (0.05-3.16)	13.73 (9.43-19.03)	4.38 (3.51-5.46)

	*p *value		0.813	≤0.001*∗*	≤0.001*∗*

3rd^#^	18.5-24	202	1.44 (0.02-3.91)	13.61 (9.74-18.17)	3.84 (2.85-5.02)

	24-28	277	1.66 (0.04-3.76)	13.51 (8.85-18.19)	3.94 (3.05-5.08)

	≥28	117	1.60 (0.31-3.88)	12.41 (8.78-17.02)	3.97 (2.91-5.05)

	*p *value		0.319	≤0.001*∗*	0.338

*∗ P *< 0.05, Kruskal–Wallis test. ^#^BMI was divided to normal weight, overweight, and obesity, because there were no underweight women in the third trimester.

**Table 4:** *β* (95% confidence interval) of TSH, FT4, and FT3 among BMI categories.

Trimesters	BMI (kg/m^2^)	TSH	FT4	FT3
1st	<18.5	-0.052 (-0.188,0.083)	0.165(-0.240,0.570)	-0.064(-0.153,0.026)

	18.5-24	Ref.	Ref.	Ref.

	≥24	0.185 (0.026,0.345)	-0.325 (-0.804,0.154)	0.145 (0.040,0.251)

	*p* value^#^	0.014*∗*	0.092	≤0.001*∗*

2nd	<18.5	0.02 3(-0.137,0.182)	0.368 (-0.090,0.826)	-0.105 (-0.203,-0.008)

	18.5-24	Ref.	Ref.	Ref.

	≥24	0.012 (-0.071,-0.094)	-0.341 (-0.578,-0.104)	0.132 (0.081,0.182)

	*p* value^#^	0.813	0.001*∗*	≤0.001*∗*

3rd	18.5-24	Ref.	Ref.	Ref.

	24-28	0.049 (-0.119,0.217)	-0.419 (-0.829,-0.008)	0.097 (-0.002,0.197)

	≥28	0.070 (-0.142,0.282)	-1.040 (-1.558,-0.522)	0.062 (-0.064,0.188)

	*p* value^#^	0.963	≤0.001*∗*	0.280

Ref.: reference category. *∗P *< 0.05, ^#^*p *value for trend. *P* value for trend, ratio, and 95% CI were calculated by multiple liner model, adjusted for residence (rural, urban), age (years), gestational age (weeks), and parity (primipara, multipara).

**Table 5:** Relationship between BMI and thyroid dysfunction in the logistic regression analysis.

	*n*	Rude OR (95% CI)	*p* value	Adjusted OR(95% CI)	*p* value
Subclinical hypothyroidism	79	0.993(0.929-1.062)	0.836	0.991(0.917-1.072)	0.825

Isolated hypothyroxinemia	78	1.107(1.042-1.177)	≤0.001	1.081(1.007-1.161)	0.031*∗*

*∗P* < 0.05. Odds ratio (OR) and 95% confidence interval (CI) were estimated using binary logistic regression models, adjusted for residence (rural, urban), age (years), gestational age (weeks), and parity (primipara, multipara).

## Data Availability

The data used to support the findings of this study are available from the corresponding author upon request.
